# Memoranda Medica; or Note-Book of Medical Principles

**Published:** 1861-01

**Authors:** 


Art. VII.—*Memoranda Medica, or Note-Book of Medical Principles. Being a Concise Syllabus of Etiology, Semeiology, General Pathology, Nosology, and General Therapeutics. With a Glossary. For the Use of Students.* By Henry Hartshorne, A.M., M.D., Professor of Theory and Practice of Medicine in the Medical Department of Pennsylvania College, etc., etc., etc. Ὠφελεειν ἢ μη βλάπτειν. "*To heal, or not to harm.*" 12mo., pp. 190. J. B. Lippincott & Co.: Philadelphia, 1860.

It is difficult to convey, by any verbal description, a correct idea of these "memoranda." Although in their conception they can scarcely be esteemed original, they are eminently so in their execution. They are intended, the author remarks, to occupy "a position intermediate between the professor's skeleton synopsis or syllabus and the elaborate treatise or text-book." If, however, a syllabus may be defined properly "an abstract or compendium"—varying within certain limits, in respect to its scope and fullness—the appellation may then with strict propriety be applied to the work before us. It can, in fact, be considered in no other light than as simply a syllabus of the fundamental and introductory portion of the course of lectures delivered by the author in the department of the theory and practice of medicine—presenting a brief, lucid, and accurate outline of medical principles.

In defining the work of Dr. Hartshorne a simple syllabus of the institutes of medicine, we desire not to be understood as in the slightest degree depreciating it. It is, in fact, so defined by the author himself, on the title-page. We would remark that we consider the talents that are required for the preparation of a good syllabus to be of no contemptible character. Besides a perfect familiarity with the subjects of which it professes to be a compendium, and with their relationship and co-ordination, there must be, also, a facility in condensation, with a proper regard to perspicuity, which it will be found is possessed but by very few, comparatively speaking.

The work before us—no matter what may be the appellation given to it—is unquestionably a most interesting one, and one which, within its appropriate scope, is most ably executed. It exhibits a very fair view of the more important points relating to the subjects embraced in it, all of which are set forth with great conciseness, but, at the same time, with the utmost clearness and accuracy. We have seldom indeed met with, in any medical work, a series of definitions which, like those that appear in the memoranda of Dr. Hartshorne, convey, in as few words, so full and definite an idea of the subjects to which they refer. The work before us furnishes precisely such a manual as may be used with decided profit by the student, and can scarcely, under any circumstances, be made an improper use of by him. Even those who have passed through their novitiate, and already entered upon the practice of their profession, may derive from a perusal of it both pleasure and instruction. The work contains, in fact, a greater amount of most excellent matter than its size would, at first sight, seem to indicate. Much, however, as we are pleased with the general plan and execution of these memoranda medica, we could have desired, in respect to some few of them, greater fullness—more of those aphoristic propositions in which the author has succeeded so admirably in condensing such an amount of valuable matter without in the slightest degree impairing its clearness. In relation also to certain points, in connection more especially with the department of general pathology, which are entirely overlooked by Dr. Hartshorne, we could have wished for the insertion of a memorandum or two. We say this in no fault-finding spirit. It is from our high appreciation of the excellence of what the author has done, that we feel a regret that he has not done more.

We may be permitted to remark that we doubt the correctness of the rendering which Dr. Hartshorne has given to the sentence from Hippocrates, which he has placed on his title-page as a motto. Is not, we would respectfully ask, its true meaning "to *do good* rather than *merely not to injure?*" Implying, in other words, that it is the duty of the physician positively to benefit his patient rather than simply to avoid doing him harm. That the Greek ἢ has often the sense of "rather than," is a fact of which every scholar is perfectly aware.

It is to be hoped that Dr. Hartshorne may receive sufficient encouragement, by the favorable reception of the volume under notice, to induce him to carry out his intention of preparing parallel memoranda upon the subjects of nosography, special pathology, and the practice of medicine.

